# Determinants and prognostic implications of Cardiac Troponin T measured by a sensitive assay in Type 2 Diabetes Mellitus

**DOI:** 10.1186/1475-2840-9-52

**Published:** 2010-09-15

**Authors:** Jonas Hallén, Odd Erik Johansen, Kåre I Birkeland, Lars Gullestad, Svend Aakhus, Knut Endresen, Solve Tjora, Allan S Jaffe, Dan Atar

**Affiliations:** 1 Department of Cardiology, Oslo University Hospital, Oslo, Norway; 2 Faculty of Medicine, University of Oslo, Oslo, Norway; 3 Medical Department, Vestre Viken, Asker and Baerum Hospital, Rud, Norway; 4 Department of Endocrinology, Oslo University Hospital, Aker, Oslo, Norway; 5 Department of Cardiology, Oslo University Hospital, Rikshospitalet, Oslo, Norway; 6 Department of Laboratory Medicine, Oslo University Hospital, Oslo, Norway; 7 Cardiovascular Division, Department of Medicine, Mayo Clinic, Rochester, USA

## Abstract

**Background:**

The cardiac troponins are biomarkers used for diagnosis of myocardial injury. They are also powerful prognostic markers in many diseases and settings. Recently introduced high-sensitivity assays indicate that chronic cardiac troponin elevations are common in response to cardiovascular (CV) morbidity. Type 2 diabetes mellitus (T2DM) confers a high risk of CV disease, but little is known about chronic cardiac troponin elevations in diabetic subjects. Accordingly, we aimed to understand the prevalence, determinants, and prognostic implications of cardiac troponin T (cTnT) elevations measured with a high-sensitivity assay in patients with T2DM.

**Methods:**

cTnT was measured in stored, frozen serum samples from 124 subjects enrolled in the Asker and Bærum Cardiovascular Diabetes trial at baseline and at 2-year follow-up, if availabe (96 samples available). Results were analyzed in relation to baseline variables, hospitalizations, and group assignment (multifactorial intensive versus conventional diabetes care for lowering CV risk).

**Results:**

One-hundred thirteen (90 %) had detectable cTnT at baseline and of those, 22 (18 % of the total population) subjects had values above the 99th percentile for healthy controls (13.5 ng/L). Levels at baseline were associated with conventional CV risk factors (age, renal function, gender). There was a strong correlation between cTnT levels at the two time-points (r = 0.92, p > 0.001). Risk for hospitalizations during follow-up increased step-wise by quartiles of hscTnT measured at baseline (p = 0.058).

**Conclusions:**

Elevations of cTnT above the 99th percentile measured by a highly sensitive assay were encountered frequently in a population of T2DM patients. cTnT levels appeared to be stable over time and associated with conventional CV risk factors. Although a clear trend was present, no statistically robust associations with adverse outcomes could be found.

## Introduction

The majority of deaths in patients with type 2 diabetes mellitus (T2DM) are due to cardiovascular (CV) disease. Although hyperglycemia, the defining feature of T2DM, is closely associated with microvascular and macrovascular complications[1], studies have failed to demonstrate that glucose lowering *per se *reduces the risk of macrovascular events [2-4]. Thus, current understanding suggests that broad, multiple intervention strategies including lifestyle changes, aggressive blood pressure and lipid lowering, in addition to glucose control, are necessary for mitigating morbidity and mortality related to macrovascular disease [5,6]. Consistent with this concept, the prospective, randomized Asker and Bærum Cardiovascular Diabetes (ABCD) trial on 120 T2DM subjects found that structured care encompassing a comprehensive and intensive preventive strategy reduced the primary efficacy outcome of change in the estimated 10-year absolute risk for fatal coronary heart disease at 2 years compared to conventional care [7].

The cardiac troponins T and I are extremely sensitive and specific biomarkers of myocardial necrosis and crucial components of the diagnosis of myocardial infarction [8,9]. The recent emergence of several high-sensitivity assays has shown that cardiac troponins can be chronically elevated in response to CV comorbidities and that they confer important prognostic information. The presence of T2DM is a known predictor of elevated cardiac troponin T in the general population with contemporary assays [10], but the prevalence, determinants and prognostic implications of cardiac troponin T elevations measured by a high-sensitivity assay in T2DM subjects have not been investigated. Furthermore, it is unknown whether troponin levels are lowered by aggressive CV risk-factor modification in this patient group. Accordingly, in the present post hoc analysis of the ABCD study, we attempted to define the distribution and determinants of cardiac troponin T levels in patients enrolled in the trial at baseline, and investigate whether the structured, intensive care delivered in the ABCD trial results in lower cardiac troponin T values after 2 years of follow-up. In addition, we wanted to tentatively explore whether cardiac troponin T levels were associated with hospitalizations during follow-up.

## Methods

### Setting, design and participants

**Figure 1:**
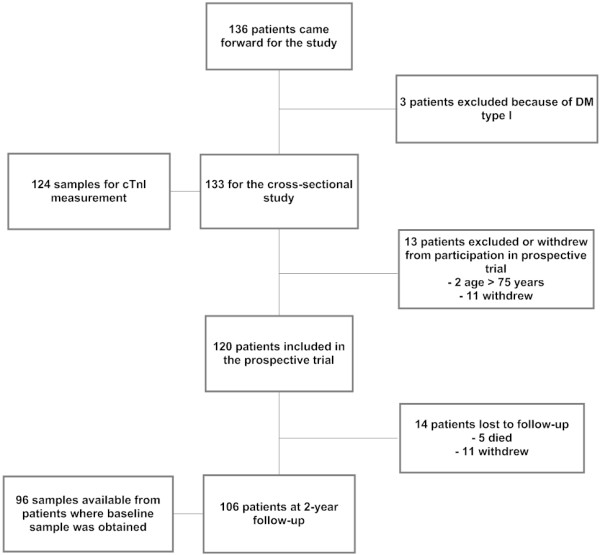
**Flow chart showing number of patients included for cross-sectional and prospective investigation and reasons for exclusion; and the number of patients who had troponin T measured at the two time points**.

We performed a post hoc analysis of the ABCD study conducted at Vestre Viken, Asker and Baerum Hospital, Rud, Norway from 2002 to 2006. The study enrolled 133 subjects with T2DM for a cross-sectional investigation of the prevalence of coronary artery disease. Exclusion criteria were clinically significant peripheral (defined as amputation-threatening ischemia) or cerebral artery disease or type 1 DM (the presence of autoantibodies towards pancreatic beta cells and insulin initiation within one year since the diagnosis of T2DM). Participants underwent clinical examination, 24 hour Holter monitoring and blood pressure measurements, stress electrocardiogram, and testing of ventilatory oxygen uptake, in addition to blood and urine sampling. 120 of the original 133 participants had ≥ 1 CV risk factor and agreed to be part of a prospective, randomized controlled trial of intensive versus usual care in T2DM subjects. Figure 1 provides an overview of the inclusion and exclusion of patients. Individuals included in the prospective trial were referred for voluntary stress echocardiography and invasive coronary angiography. Details of the design and results of the cross-sectional and prospective studies have been published [7,11]. All participants gave written informed consent and the studies were conducted in accordance with the Helsinki Declaration and approved by the Regional Ethics Committee and the Norwegian Data Inspectorate.

### Data collection and procedures

Cardiac troponin T was measured in blood samples at baseline (n = 124) and 2-year follow-up (n = 96), if available. Samples were stored at - 80°C for 4-8 years and measured on the Roche Diagnostics (Basel, Switzerland) Cardiac troponin T assay by the electrochemiluminescence method. The limit of the blank is 3 ng/L, the limit of detection 5 ng/L, and the 99th percentile was determined to be 13.5 ng/L in 616 apparently healthy volunteers [12]. Assays were performed by personnel unaware of the patient's identity or other characteristics. Details regarding sampling strategies and assay characteristics for the laboratory determinations of the other serum markers have been described previously [7,11].

Details of all procedures and clinical tests have been published previously [7,11]. In brief, for the exercise test we used a modified maximum symptom-limited 1-min incremental exercise test on an electrically braked cycle ergometer (Siemens-Elema, Germany). The patients maintained a constant pedaling-rate of 60 rotations per minute. Simultaneous haemodynamic monitoring was performed and symptoms of chest pain were recorded during exercise. A 12-lead Likar-Mason modified electrocardiogram was continuously sampled every 2 ms (500 Hz sampling rate) and converted to digital form with a 12-bit resolution. Standard ST-segment depression criterion (> 1 mm [0.1 mV]) or additional horizontal or down-sloping ST-segment depression at end exercise was used for assessment of the ECG.

Dobutamine stress echocardiography was performed using a staged protocol with dobutamine infusions up to 40 μg/kg/min in increments of 3 minutes with atropine 0,25-1,0 mg added when needed to obtain peak heart rate [13]. Peak stress was defined as either of: new or worsened left ventricular regional wall motion abnormality, HR > 220 - age (years), blood pressure > 200/110 mmHg, or patient discomfort. Ultrasound cine loops of left ventricle imaged from 3 apical and 2 parasternal imaging planes were obtained (Vivid 7 or Vivid 5 scanners, GE Vingmed Sound, Horten, Norway) at rest, at 10 and 20 μg dobutamine/kg/min and at peak stress, and transferred for off-line analysis. Left ventricular wall motion analysis was performed by a blinded experienced observer. Wall motion score index was assessed by use of a 16 segments model of the left ventricle [14]. Reversible myocardial ischemia was defined as new or progressing wall motion abnormality during test in > = 1 segment.

Coronary angiography was performed using standard Judkins' technique, with a percutaneous radial or femoral approach using 6F diagnostic catheters (Cordis Corporation, Miami, FL, US) and the water-soluble, non-ionic, dimeric contrast medium iodixanol (Visipaque 320 mg/mL; Amersham Health, Oslo, Norway). The angiograms were performed after routine referral to the catheterization lab (194 ± 94 days after enrolment in the study) and were independently analyzed semiquantitatively by two experienced cardiologists. Stenosis > 50% of lumen diameter was defined as main vessel disease and patients were categorized as 0-, 1-, 2- or 3-vessel disease. Stenosis of the left main coronary artery of greater than 50% of lumen diameter was considered to be 2-vessel disease. Inter-observer variability of angiographic classifications was 4.9 %. The operators were blinded to randomization and hscTnT levels.

Hospitalizations were defined as hospital stays = > 24 hours and were adjudicated by the principal investigator (OEJ) and study nurse at each follow-up visit and by review of patient charts. Estimation of 10-year risk for coronary artery disease was performed using the UK Prospective Diabetes Study risk algorithm [15], a diabetes-specific model that incorporates HbA1c, systolic blood pressure total and HDL cholesterol, atrial fibrillation, age, gender, ethnic group, smoking status and duration of diabetes.

### Statistical analysis

Continuous variables are presented as means with standard deviations or geometric means with 95^th ^percentiles as appropriate (median and interquartile range for cardiac troponin T), and categorical variables as proportions. Groups were defined by gender-specific quartiles of cardiac troponin T at baseline or follow-up. Statistical comparison of groups was performed by one-way ANOVA (continouous variables) or chi-square tests (categorical variables). Tests for trends were performed by linear regression analysis for continuous variables (assigning the median cardiac troponin T value for each quartile category to each person in that category as the independent variable in the model) and Cochran-Armitage trend test for categorical variables. For comparison of cardiac troponin T values between groups (i.e. men vs. women, intensive vs. conventional care, and baseline vs. 2-year samples), Wilcoxon signed-rank or Wilcoxon rank-sum tests were employed as appropriate. Reported correlation coefficients are Spearman rank correlations. Event rates (i.e. hospitalizations) are presented as proportions. A p-value of less than 0.05 (two sided) was considered to indicate statistical significance. All statistical analyses were performed on SPSS (version 16.0, SPSS Inc., Chicago, Ill, US)

## Results

### Distribution of troponin T values and patient characteristics

**Figure 2:**
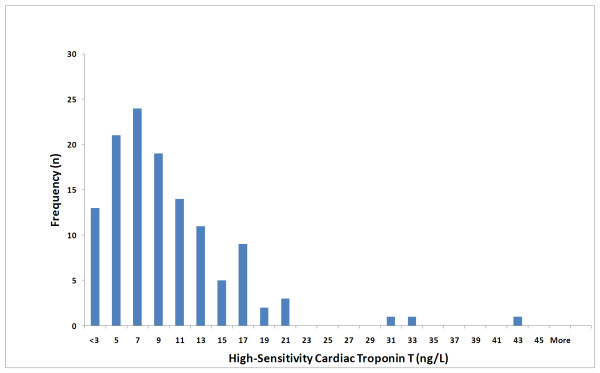
**Distribution of cardiac troponin T by the high-sensitivity assay in patients with type 2 diabetes mellitus**.

**Figure 3:**
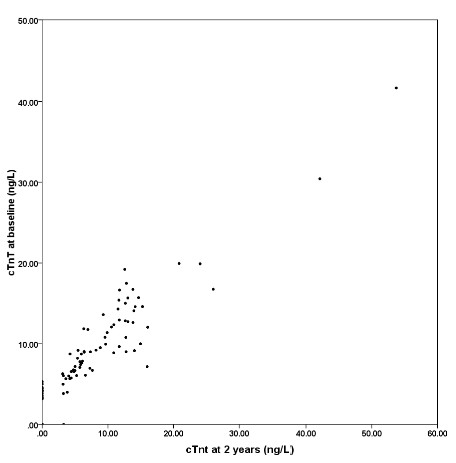
**Scatter plot of cardiac troponin T values at baseline and 2 years**.

At baseline, median (interquartile range) troponin T for all samples were 7.3 (6.1) ng/L. For patients in whom two measurements were available, median (interquartile range) troponin T values were 7.5 (7.7) ng/L and 5.8 (11.8) ng/L at baseline and follow-up, respectively (p < 0.001 for change). Hundred -and-eleven patients (90%) had measureable levels of troponin T at baseline and of those, 22 patients had values above the 99th percentile of a reference population. Troponin T levels were higher in men than in women (median, 8.2 ng/L vs. 5.6 ng/L; p < 0.01). The distribution of troponin T concentrations at baseline is shown in figure 2. Patient characteristics according to gender-specific quartiles of baseline troponin T levels are shown in table 1. Troponin T levels were associated with several conventional risk factors (increasing age, male gender, renal impairment and duration of diabetes). There was a strong correlation between troponin T values at baseline and follow-up (rho = 0.92; p < 0.001) (figure 3).

**Table 1:** Comparison of variables between subjects stratified by cardiac troponin T levels

Distribution (n = 124)	Quartile 1 (n = 31)	Quartile 2 (n = 31)	Quartile 3 (n = 31)	Quartile 4 (n = 31)	P-value*	P-value for trend†
Cardiac troponin T (ng/L), range						
Men	0.00 - 5.73	5.74 - 8.19	8.20 - 12.62	12.62 - 41.69		
Women	0.00 - 3.00	3.01 - 5.64	5.65 - 8.48	8.49 - 30.42		
**Demographic and clinical data**						
Age (years, mean, SD)	52 (10)	58 (8)	62 (9)	62 (9)	< 0.001	< 0.001
BMI (kg/h2, mean, SD)	30 (7)	30 (4)	31 (5)	29 (6)	0.586	0.612
24 h systolic BP (mm/Hg, mean, SD)	127 (14)	133 (10)	136 (12)	133 (18)	0.079	0.124
24 h diastolic BP (mm/Hg, mean, SD)	76 (9)	79 (7)	80 (7)	76 (9)	0.231	0.839
S-L voltage criteria (mm, mean, SD)	21 (8)	22 (8)	20 (7)	21 (9)	0.575	0.908
Ejection fraction (%, mean, SD)	63 (10)	63 (8)	63 (5)	64 (8)	0.907	0.546
V02 max (liters/minute, mean, SD)	2.3 (0.6)	2.1 (0.6)	2.1 (0.7)	1.9 (0.7)	0.188	0.036
**Medical history**						
Duration of diabetes (years, mean, SD)	5 (5)	7 (6)	5 (6)	10 (8)	0.008	0.005
Coronary artery disease	3 (10%)	5 (16%)	4 (13%)	3 (10%)	0.841	0.902
Hypertension	18 (58%)	21 (68%)	21 (68%)	24 (77%)	0.448	0.124
Current smoker	4 (13%)	4 (13%)	3 (10%)	3 (10%)	0.956	0.612
**Laboratory determinations**						
BNP (pmol/L, geometric mean, 95^th ^percentile)	12 (8-18)	8 (6-12)	12 (8-17)	16 (9-27)	0.211	0.163
Cholesterol (mmol/L, mean, SD)	4.9 (1)	4.9 (1)	5.2 (1)	4.8 (1)	0.492	0.740
CRP (g/dL, geometric mean, 95^th ^percentile)	3 (2-4)	2 (2-3)	3 (2-4)	2 (2-3)	0.530	0.554
Hb1Ac (%, mean, SD)	7.4 (1)	7.4 (2)	7.4 (2)	7.8 (2)	0.641	0.254
eGFR (Cockroft-Gault) (ml/min, mean, SD)	137 (55)	120 (37)	115 (37)	102 (33)	0.011	0.001
Microalbuminuria (ug/min, geometric mean, 95^th ^percentile)	15 (11-22)	14 (11-19)	22 (15-30)	18 (13-25)	0.285	0.306
**Exercise ECG (n = 124)**						
Number of patients	31	31	31	31		
Ischemia	9 (29%)	8 (26 %)	10 (32%)	13 (42%)	0.559	0.231
**Angiography (n = 85)**						
Number of patients	19	20	21	25		
Coronary disease	2 (9%)	6 (30%)	5 (24%)	9 (36%)	0.273	0.098
**Stress echocardiography (n = 87)**						
Number of patients	20	22	22	23		
Reversible ischemia	3 (15%)	6 (27%)	6 (27%)	7 (30%)	0.674	0.283

* P-value is for comparison between groups and derived from one-way ANOVA (contionuous variables) or Chi-Square test (categorical variables).

† For continuous variables, the test for trend is based on assigning the median troponin T value for each quartile category to each person in that category and then treating these four values as a continuous, independent variable in a linear regression model. For categorical variables, the test is based on the Cochran-Armitage trend test.

SD = standard deviation; BMI = body-mass index; BP = blood pressure; S-L = Sokolow-Lyon; BNP, = Brain-natriuretic peptide; CRP = C-reactive protein; eGFR = estimated glomerular filtration rate.

### Troponin T values according to CV risk, hospitalizations and treatment strategy

**Figure 4:**
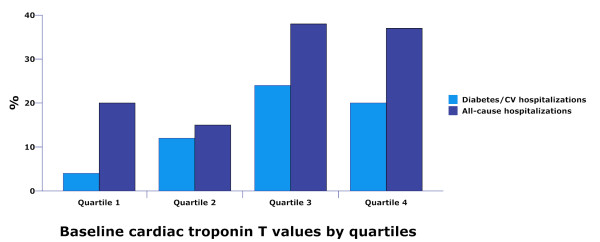
**Bar charts of all-cause and diabetes-related hospitalizations according to cardiac troponin T levels stratified by quartiles at baseline**. P-value for trend is 0.058 (all-cause) and .055 (diabetes-related). CV = cardiovascular

The number of patients with all-cause and diabetes/CV-related hospitalizations increased in a step-wise fashion by quartiles of troponin T values at baseline (figure 4). Patients without hospitalizations experienced a statistically significant larger drop in median (interquartiles range) troponin T values during follow-up compared to patients hospitalized for any reason (- 2.0 ng/L [3.7] versus - 1.2 [4.7] ng/L, p = 0.019) and/or for diabetes-related reasons (- 1.8 [3.5] ng/L versus - 1.1 [6.4] ng/L, p = 0.032).

No differences were seen in troponin T levels at baseline (7.2 ng/L [8.0] versus 7.7 ng/L [8.0] for the intensive and conventional strategy, respectively), at 2 years (5.7 ng/L [12.7] versus 5.9 ng/L [11.7]), or in the change in values between the structured and conventional treatment groups.

## Discussion

In a relatively young and healthy T2DM population we found that circulating troponin T were measureable in 90 % of individuals by the new high-sensitivity assay, and that a substantial proportion had levels above the 99th percentile of a reference population. Troponin T values were associated with several conventional risk factors for CV disease, and concentrations of troponin T remained largely stable throughout the follow-up period. These observations imply that in this patient population, troponin T release reflects underlying and chronic pathophysiological processes. Thus, troponin T may be a useful surrogate marker to define risk among subjects with T2DM, and may facilitate early identification of patients with subclinical CV disease and other high-risk individuals.

Using the older and less sensitive conventional assay, a previous analysis observed elevated troponin T values in about 0.7 % of the general population [10]. Elevations were associated with established CV disease or high-risk phenotypes including T2DM. This is consistent with the substantial proportion of T2DM patient with troponin T elevations above the 99^th ^percentile found in the present dataset. Our results also support and extend previous investigations of troponin T values in populations with established CV disease [16-18]. In these conditions, mechanisms of troponin release is thought to include mismatch between oxygen supply and demand, inflammation, apoptosis, and myocardial strain [19,20]. We found a consistent trend of higher proportions of patients with coronary artery disease indicated by coronary angiography, or positive exercise and stress-echocardiography tests, in quartiles of increasing troponin T values. However, none of these differences amounted to statistical significance which could indicate that our study was underpowered to establish these relationships in a statistically robust fashion. The finding of a negative correlation between ventilatory oxygen uptake and troponin T values fits with the well-established association between CV disease and aerobic capacity [21,22], and may reflect the cardioprotective properties of physical exercise.

We were not able to demonstrate that intensive, structured care translated into lower troponin T values, despite the fact that such interventions improved risk profiles (lipds, blood pressure, hyperglycemia) as described in the primary report from the ABCD trial [7]. Possibly, a longer follow-up of patients is needed to detect such differences in troponin T levels. Overall, there was a small, albeit highly statistically significant reduction in troponin T levels during the follow-up. This trend may reflect an analytical basis, or, alternatively, the efficacy of therapeutic interventions in the population as a whole (as opposed to the structured care group alone).

The follow-up at 2 years allowed us to tentatively probe the prognostic properties of troponin T, although the post hoc design restricts interpretation and some important limitations should be recognized: First, according to the study protocol, hospitalizations were only classified as all-cause or diabetes/CV-related. It would have been instructive to also consider only CV hospitalizations. Second, adjustments for relevant covariates were not possible due to the low number of patients and endpoints. In light of these limitations no inferences can be drawn on the basis of the trend of increasing hospitalizations across quartiles of increasing troponin T values, even though the prognostic value of troponin T that these numbers allude to are in agreement with previous reports from other much larger populations [16,17]. Interestingly, patients with no hospitalizations experienced a statistically significant larger drop in troponin T values compared to patients who were hospitalized. This is an intriguing corollary to the general trend of very stable elevations in the long term and could be taken to suggest that even small alterations in troponin T levels are indicative of changes in the underlying cardiac status of patients [18].

The larger context of the present study is the increasing use of circulating biomarkers for diagnostic and prognostic purposes - as well as to directly inform treatment decisions - in many fields in medicine. Cardiovascular diseases and associated conditions like diabetes are no exceptions, as new studies are emerging defining novel biomarkers as well as exploring the role of well-known markers in new contexts [23,24]. In response to these developments benchmarks have been proposed for evaluation of the clinical usefulness of biomarkers [25]. The cardiac troponins are well-established biomarkers recognized as providing diagnostic and prognostic information in both acute and chronic conditions. Recent studies have shown that the novel high-sensitivity troponin assays further improve diagnostic and prognostic performance compared to the conventional assays [16,17,26]. Our study extends these findings to a diabetic population and suggests some clinical implications. They alert the clinical community that individuals with T2DM - even if fairly young and without much apparent comorbidity - have a substantial probability of having a chronically elevated troponin T value with the new high sensitivity assay. In terms of diagnostic testing, these observations underline the importance of the rising and/or falling pattern, as suggested by the guidelines, to identify those who are having acute myocardial infarctions in this population [8,27]. For prognostic purposes, the relationship we observed of troponin T with future hospitalizations and several other risk factors could point to a future role of the biomarker as a risk stratification tool in this context, but the data presented here should be considered preliminary and need to be validated in a larger cohort where adjustment can be made for other established risk factors.

Some important limitations with this investigation have already been mentioned and others should also be considered. This was a post hoc study performed in a relatively small cohort and due to the low absolute number of patients and events, we were not able to explore our data in multivariable regression models. Clearly, this represents a major weakness of our study. Yet, our findings fit well with the current understanding of the distribution and prognostic properties of troponins in populations with established, or at high risk for, CV disease (14, 15, 16). It is likely that some of the troponin T elevations seen in our population compared to the reference population are related to increased age, although increases with this assay were not reported as part of the assay validation. In any case, this does not restrict interpretation of the results, as our population is representative of a real-life T2DM population; and that regardless of cause troponin-release confers a higher risk of CV events and death. Measurements of baseline and 2-year troponin T samples were performed at different time-points, which increase the analytical variability and may explain the slight fall in troponin T levels during follow-up.

In conclusion, in a population of T2DM patients, we found that almost 1 in 5 had troponin T values above the 99^th ^percentile of a reference population when measured by a highly sensitive assay. Levels of troponin T were stable over time, associated with conventional risk factors, and displayed a graded relationship with hospitalizations although this trend was not statistically significant.

## Competing interests and funding

Roche Diagnostics (Basel, Switzerland) provided reagents for troponin T assays. JH and OEJ were supported by grants from the South-Eastern Norway Regional Health Authority. JH also received support from Aker University Hospital Research Foundation and Center for Heart Failure Research, Oslo, Norway. ASJ has consulted over time for most of the major diagnostic companies. OEJ is an associated post-doctoral researcher at Vestre Viken, Asker and Bærum Hospital (Rud, Norway) and JH is a PhD student at Oslo University Hospital and the University of Oslo, but both are employees of Boehringer Ingelheim.

## Authors' contributions

JH conceived of and designed the study, performed the cardiac troponin T measurements, performed all statistical analysis and wrote the first draft of the manuscript. OEJ was principal investigator of the ABCD study and was responsible for data collection and diagnostic tests. KB and LG supervised the ABCD study and ASJ and DA supervised this substudy. SA and KE participated in collection of data and performed diagnostic tests. SJ performed the cardiac troponin T measurements. All authors contributed to the design of the study, interpretation of the results, and critical revision of the manuscript. All authors have read and approved the final manuscript.
